# Look Behind Me! Highly Informative Picture Backgrounds Increase Stated Generosity Through Perceived Tangibility, Impact, and Warm Glow

**DOI:** 10.3389/fpsyg.2022.800199

**Published:** 2022-04-06

**Authors:** Marta Caserotti, Martina Vacondio, Maya Maze, Giulia Priolo

**Affiliations:** ^1^Department of Developmental Psychology and Socialization, University of Padua, Padua, Italy; ^2^Department of Cognitive Psychology, University of Klagenfurt, Klagenfurt, Austria; ^3^Department of General Psychology, University of Padua, Padua, Italy; ^4^Department of Psychology and Cognitive Sciences, University of Trento, Rovereto, Italy

**Keywords:** prosocial behavior, donation, tangibility, visual information, background, impact, warm glow, COVID-19

## Abstract

In this study, we investigated whether background information of a visual charity appeal can influence people’s motivation to donate and the hypothetical amount donated. Specifically, participants were presented with a charity appeal to help a local hospital respond to the Coronavirus Disease-19 (COVID-19) emergency depicting a man sitting on a bed in a hospital room. The number of visual details (i.e., medical equipment) depicted in the background was manipulated according to three conditions: (1) “High information” condition (i.e., a room full of medical equipment), (2) “low information” condition (i.e., room with few pieces of medical equipment), and (3) “no information” condition (i.e., non-contextual background). We investigated whether the number of visual background details would have increased the tangibility of the cause measured as the hospital’s adequate preparedness to deal with the COVID-19 emergency and severity of the patient’s medical conditions. We also investigated whether increased tangibility, elicited by a higher amount of background information, would heighten participants’ perceived impact of their donation and warm glow, which in turn would have led to increased motivation to donate and the amount donated. We found no significant direct effect of condition on the donated amount. However, path models revealed that more background information positively influenced participants’ motivation to donate and the amount donated indirectly through increased tangibility, impact, and warm glow. Finally, we showed that a higher risk perception of COVID-19 was associated with higher donations. Results are discussed in line with relevant literature.

## Introduction

The recent Coronavirus Disease-19 (COVID-19) pandemic brought to the fore the fragilities of several health systems, undermining the stability of health agencies and governments around the world. To support medical facilities burdened by the emergency, the governments themselves, as well as charities, and non-profit organizations, increased their effort to raise funds to address the health and social emergency that was and still is pervasive. In 2020, Americans alone have responded to such calls by donating more than 42 billion to health-related causes (Giving USA, 2021), funds that were critical in supporting important health and social projects. Hence, this situation shed light on the pivotal role of private donations in supporting distressed communities, increasing the necessity and urgency to better understand how to encourage and increase donations to deal with both sudden and chronic emergencies.

Although a variety of studies have investigated the factors that may contribute to a successful donation appeal, most have focused on the role of the donation recipient characteristics (in both visual and textual appeals), leaving out, to the best of our knowledge, information related to a contextual background. Therefore, this study is aimed at filling this gap by investigating the role played by visual background information depicted in a charity appeal in shaping donation behaviors.

Previous literature on facial expressions of donation recipients, has demonstrated how both distressed (e.g., Small and Verrochi, 2009; Cao and Jia, 2017; Jang et al., 2019) and happy (Zemack-Rugar and Klucarova-Travani, 2018) expressions, can elicit empathy in donors and thus increase their donations. Further research demonstrated that people tend to donate more to identifiable victims, i.e., presented through personal details that identify them (Small et al., 2007), rather than to a greater or equal number of unidentified or statistical victims (Schelling, 1968; Jenni and Loewenstein, 1997; Small and Loewenstein, 2003). This effect also holds when a single identified victim is compared to a group of equally identified victims (Kogut and Ritov, 2005; Small et al., 2007; Kogut, 2011).

The positive effect of a single identified victim on pro-social behavior has been explained in terms of increased tangibility (Cryder and Loewenstein, 2010). Tangibility refers to the degree of specificity and concreteness of the mental representation of a situation. It depends on the richness of details used to describe the situation or the way those details are processed. Tangibility positively impacts generosity through three interrelated causal mechanisms (Cryder and Loewenstein, 2010). First, providing tangible information about the charity and the project that will benefit from the donation increases perceived impact (i.e., donors’ perception of how much their contribution can concretely help the beneficiary; Erlandsson et al., 2014, 2015), which in turn leads to greater prosocial behavior (Cryder and Loewenstein, 2010; Cryder et al., 2013). Second, vivid and tangible information with high imaginability boost generosity through increased emotional responses toward the recipients (Slovic, 2007; Cryder and Loewenstein, 2010; Cryder et al., 2013). Finally, a higher perceived impact elicited by giving to a tangible cause can also increase donors’ “warm glow” (i.e., anticipated and experienced good feelings associated with doing something good for others; Andreoni, 1990; Cryder and Loewenstein, 2010; Dickert et al., 2016).

In general, perceived impact of a donation and warm glow have been both identified as core motivations of prosocial behaviors and charitable giving (Andreoni, 1990; Duncan, 2004; Dunn et al., 2008; Cryder et al., 2013; Erlandsson et al., 2014, 2015; Västfjäll et al., 2015). For instance, when overhead costs (i.e., administrative expenses of charitable organizations) are perceived as high (Sargeant and Woodliffe, 2007; Caviola et al., 2014), perceived donation impact drops thus consequently reducing motivation to donate. Similarly, a warm glow has been found to motivate people to act prosocially by positively impacting donors’ short-term affective reactions (Konow, 2010). Specifically, self-focused feelings (i.e., warm glow) have been found to directly influence the motivation to donate, but not always the amount donated (Dickert et al., 2011).

Since tangible and vivid information about the cause or the recipient can increase prosocial behaviors, it is plausible that visual information depicted in the background of a visual charity appeal can influence people’s willingness to donate through increased tangibility too. Nevertheless, studies focusing on background information in the prosocial domain are relatively scarce. A recent study by Choi et al. (2020) tested the influence of background on charitable giving. However, this study focused on the concordance between the positive or negative emotions generated by charity appeal messages (i.e., text and images) and the background color used (i.e., blue and orange), showing that the contrast between the two increases donations. Notably, this study examined a solid (or context-free) background, namely, a color background that lacks any kind of pattern or specific contextual information.

On the contrary, the role of the contextual background has been widely studied in marketing but results are mixed. In e-commerce, websites’ products can be presented with a white, context-free background or with a background related to the context of the use of the target product. Some studies suggested that context-free images are preferred to contextual ones because image details derived from the background increase its complexity while decreasing liking (Winkielman et al., 2003). More recent studies, however, indicate that despite their greater complexity, contextual images can be perceived more fluently and enjoyed more, since they facilitate product recognition (Maier and Dost, 2018). Notably, the contextual background has a positive effect on product evaluation, especially, for more ambiguous products, since the greater amount of information helps to reduce the number of possible interpretations (Maier and Dost, 2018), thus eliciting more favorable attitudes toward the product (Wang et al., 2020; Wu and Li, 2021).

Hence, drawing on the abovementioned literature, the present study aims at investigating whether the number of visual details (i.e., medical equipment) depicted in the picture’s background of a charity appeal can influence the motivation to donate and the amount donated. We investigated whether a higher amount of information (vs. no or low information) depicted in the background of a visual charity appeal should increase people’s perceived tangibility of the cause and in turn their motivation to donate and the amount donated. In addition, we inquired whether this relationship could be mediated by higher perceived donation impact and warm glow.

## Methods

### Participants

We recruited 474 American respondents *via* MTurk (Paolacci et al., 2010) with human intelligence task Approval Rate greater than 95% and paid them 0.10$. TurkGate (Goldin and Darlow, 2013) was used to avoid multiple responses from the same participant. Participants (women = 46%; M_age_ = 38.75; *SD* = 11.35) were randomly assigned to one of three between-subject conditions (“high information” *n* = 157; “low information” *n* = 156; and “no information” *n* = 161). No significant differences in the demographics (e.g., age, gender, education, political orientation, and type of health insurance) have been found among conditions (see Supplementary Table 1). The study has been conducted under the Declaration of Helsinki and informed consent was obtained for all participants before the completion of the questionnaire.

### Design and Procedure

Data collection took place on August 18, 2020. On that day, the recorded number of COVID-19 cases in the United States was 5,377,178, while 31,678 new hospitalizations were recorded in that week only. At that point in the pandemic, the fatality rate was 3.13% (Ritchie et al., 2020).

Participants were presented with a written donation appeal for a “COVID-19 Relief Fund” to help their local hospitals best respond to the pandemic. Together with the text, the picture of a patient, with his back turned, sitting on a hospital bed was presented. The amount of medical equipment in the picture’s background was manipulated to vary the quantity of information provided according to three experimental conditions: (1) “High information”: The patient was depicted in a hospital room filled with a high amount of medical equipment; (2) “low information”: The patient was depicted in a hospital room with a low amount of medical equipment; and (3) “no information”: The patient was depicted with a white background (for more details see Supplementary Method 1).

Participants were asked to report their motivation to hypothetically donate on a 7-point scale ranging from 1 (“Not at all”) to 7 (“Very much”) and whether they wanted to make a donation (Yes/No). Those who responded “Yes” were then asked the amount they would like to donate (amount; 10$, 25$, 50$, 75$, 100$, and others). Then, they were asked to what extent they thought that their donation could make a difference (impact) and how good donating to the Relief Fund made them feel (warm glow). Responses were given on a 7-point scale from 1 (“Not at all”) to 7 (“Very much”).

Perceived tangibility was then assessed with two *ad hoc* items. Specifically, participants had to rate on a slider from − 10 (“not prepared at all”) to + 10 (“absolutely prepared”) to what extent did the local hospital depicted in the picture seem adequately prepared for the medical emergency (adequacy), and from − 10 (“not severe at all”) to + 10 (“extremely severe”) to what extent did the medical situation of the man in the picture seemed severe (severity of the patient).

Finally, the risk perception of COVID-19 was assessed by adapting two items from Caserotti et al. (2021, 2022) and Vacondio et al. (2021). Participants were asked to rate their likelihood and their family and friends’ likelihood (Likelihood) to contract COVID-19 in the next months from 1 (“extremely low”) to 7 (“extremely high”) and to what extent they perceived the virus as dangerous (seriousness) to themselves and their close ones from 1 (“not dangerous at all”) to 7 (“extremely dangerous”). Given the high internal consistency (Cronbach’s alpha = 0.86), these variables were then collapsed into a single factor called “risk.” The questionnaire ended with demographic questions.

A detailed description of the conditions and supplementary analysis is displayed in Supplementary Material.

## Results

To investigate whether a higher amount of background information (i.e., condition) would lead to increased tangibility (i.e., adequacy and severity of the patient), we ran a bivariate correlation. Next, we ran path models to test the effect of the condition on our main dependent variables (i.e., motivation and amount) mediated by tangibility and the precursors of the donation (i.e., impact and warm glow).

To conduct our analyses, we recorded our variable condition and created two dummy variables using Helmert contrasts. Dummy 1 was created to contrast the presence of information (i.e., high and low information) against none information (i.e., high information = − 1, low information = − 1, no information = 2). Dummy 2 was created to contrast high information against low information condition (i.e., high information = 1, low information = − 1, and no information = 0).

### Correlations Between the Amount of Background Information and Main Dependent Variables

We conducted a Spearman correlation between our dummy variables and continuous one, whereas a Pearson correlation was run between the continuous variables. Our findings showed that higher number of information in the background (vs. low; Dummy 2) was associated, out of the two tangibility variables, only with higher perceived adequacy of the hospital, while Dummy 1 (i.e., presence of information vs. no information) did not correlate with any of the main variables in our study (see Table 1). To confirm the effect of condition on our tangibility variables, we also ran an ANOVA. Results confirmed findings from the correlation matrix (see Supplementary Method 2). These results show that high background (vs. low) information makes participants perceive the cause as more tangible, which in our study is represented by higher perceived adequacy of the hospital to face the emergency. Moreover, we found that high adequacy was associated with higher impact, warm glow, motivation, and amount.

**TABLE 1:** Correlation between amount of background information and main dependent variables.

	1	2	3	4	5	6	7
1. Dummy 1							
2. Dummy 2	–0.003						
3. Adequacy	–0.048	0.101*					
4. Severity of the patient	0.049	0.009	0.482**				
5. Impact	0.023	0.038	0.340**	0.520**			
6. Warm glow	–0.019	0.047	0.337**	0.458**	0.628**		
7. Motivation	–0.018	0.011	0.455**	0.608**	0.669**	0.663**	
8. Amount	0.011	–0.003	0.383**	0.514**	0.485**	0.438**	0.659**

*p < 0.05, **p < 0.01.

Being in high information (vs. low information) condition did not have a significant direct association with motivation, the precursor of donations (i.e., impact and warm glow), or our main dependent variables, i.e., amount. We also ran an ANOVA to specifically test our Average Treatment Effect (ATE) and we found no difference in the donated amount between conditions [Dummy 1, *F*(2,474) = 0.03, *p* = 0.868; and Dummy 2, *F*(2,474) = 0.03, *p* = 0.968]. However, the main goal of our paper was to test the psychological mechanisms that mediated the effect of background information on prosocial decisions. Therefore, we found it essential to test these mechanisms through a mediation model (path model). Indeed, extant literature demonstrated how investigating indirect effects in the absence of a total effect (i.e., ATE) is important when the goal of an experiment is to test the psychological mechanisms behind a simple effect (Zhao et al., 2010; Hayes, 2012, 2017).

### Effect of Amount of Background Information on Motivation and Amount

To investigate potential direct and indirect effects of the amount of background information on our main dependent variables, we used Stata 14 (StataCorp, 2015) to conduct a path analysis using structural equation modeling (SEM). Due to the results of the correlation analysis, we used only Dummy 2 (i.e., high vs. low information) out of the two dummy variables created.

We first examined Path Model 1 to investigate the indirect effect of Dummy 2 on motivation and amount mediated by the adequacy and the two precursors. Due to the results observed in the correlation matrix, the severity of the patients was excluded from the model, and Dummy 2 was associated directly only with adequacy. Next, we tested the direct effect of adequacy on the precursors and the main dependent variables. Finally, we investigated the direct path of the precursors on the main dependent variables and the direct effect of motivation on the amount. Further, in line with Cryder and Loewenstein (2010) and Dickert et al. (2016), we investigated the effect of impact on a warm glow. The resulting model was not significantly worse than the fully specified model, [*X*^2^(4, *N* = 474) = 1.85, *p* = 0.763] and showed good fit indices [root-mean mean-square error of approximation (RMSEA) < 0.001, *p* = 0.959, comparative fit index (CFI) = 1.000, and Bayesian information criterion (BIC) = 10,437.7] according to Kline (2011). Results showed that participants in the high information condition perceived a higher tangibility of the cause, i.e., adequacy (*z* = 0.11, *p* = 0.013). A higher adequacy was associated with a higher warm glow (indirect effect: *z* = 0.038, *p* = 0.018) and impact (indirect effect: *z* = 0.039, *p* = 0.018), and in turn led indirectly to a higher motivation [overall indirect effect: *z* = 0.05, *p* = 0.015, 95% CI (0.02, 0.20)] and a higher amount [overall indirect effect: *z* = 0.04, *p* = 0.017, 95% CI (0.02, 0.18)]. Moreover, we showed that warm glow (*z* = − 0.03, *p* = 0.495) and impact (*z* = 0.085, *p* = 0.081) had no direct effect on the amount. However, participants who reported higher warm glow and impact reported a higher motivation that led to a higher amount (indirect effect warm glow: *z* = 0.21, *p* < 0.001; indirect effect impact: *z* = 0.32, *p* < 0.001).

**FIGURE 1:**
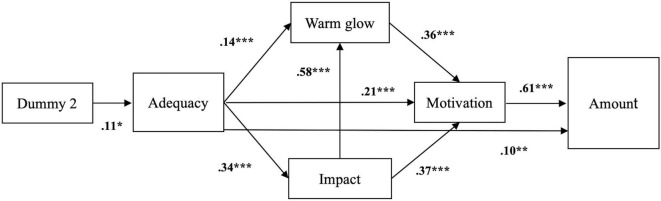
Path model testing the indirect effect of the Dummy 2 on motivation and amount, through tangibility (i.e., adequacy), warm glow and impact. Coefficients presented are standardized. *p < 0.05, **p < 0.01, ***p < 0.001.

We then removed the paths that did not show a significant effect to create a second, more parsimonious model (Figure 1). The second model tested the indirect effect of Dummy 2 (i.e., high vs. low information) on the two main dependent variables (i.e., Path Model 2). The model showed a good fit, [*X*^2^(6, *N* = 474) = 4.89, *p* = 0.558, RMSEA < 0.001, *p* = 0.934, the CFI = 1.000, and BIC = 10,428.4], and was not significantly worse than Path Model 1, [*X*^2^(2) = 3.04, *p* = 0.219]. For our main model, we also tested a path model (i.e., Path Model 2.1) where we inverted the direction of the path between impact and warm glow and the model did not differ from Path Model 2 (Goodness of fit: [*X*^2^(6, *N* = 474) = 4.89, *p* = 0.558, RMSEA < 0.001, *p* = 0.934, the CFI = 1.000, and BIC = 10,428.4)]. The results of Path Model 2 were consistent with the results of Path Model 1.

Finally, we tested a third model (i.e., Path Model 3) to control for the effect of the risk perception of COVID-19 (i.e., risk) as a covariate on our main variables. The model showed a good fit, [*X*^2^(6, *N* = 474) = 4.34, *p* = 0.631, RMSEA < 0.001, *p* = 0.953, the CFI = 1.000, and BIC = 11,920.63]. The results showed that people who perceived a higher risk also perceive a higher adequacy (*z* = 0.34, *p* < 0.001), a higher impact (*z* = 0.26, *p* < 0.001), and warm glow (*z* = 0.11, *p* = 0.006). Finally, higher risk was also associated with higher motivation (z = 0.19, *p* < 0.001) and amount (*z* = 0.11, *p* = 0.005).

## Discussion

In this study, we investigated the effect of background information depicted in a visual charity appeal on prosocial behaviors. While previous studies inquired the role of contextual background mainly in e-commerce advertisements (Maier and Dost, 2018; Wu and Li, 2021), this is among the first articles that address this issue on donation behavior.

Results show that a higher amount of information (vs. low information) depicted in the background of a visual charity appeal increased participants’ perceived tangibility of the cause. This result is in line with previous studies showing that higher amounts of textual details in written charity appeals increase the perceived tangibility of the cause (Cryder and Loewenstein, 2010; Cryder et al., 2013). Further, we show that the presence vs. the absence of contextual information does not produce *per se* a difference in perceived tangibility. This result stands between mixed findings that, in the e-commerce literature, show advantages of presenting products both with and without background (Winkielman et al., 2003; Larsen et al., 2004; Reber et al., 2004; Maier and Dost, 2018). We thus extend the literature by showing how the effect of tangibility holds also for pictorial details in the background of visual appeals. Further, in the present study, we used two *ad hoc* items to assess tangibility. Among those, only the one related to how the hospital seemed to be adequately prepared to deal with the COVID-19 emergency was found to increase along with the number of background details, while no effect was found for the perceived severity of the medical conditions of the patient depicted in the appeal. We can speculate, therefore, that our manipulation of the contextual information effectively influenced the perception of the environment in which the scene took place, but did not affect the perception of the victim since no information directly associated with him has been instead manipulated.

Results also demonstrated that the higher tangibility perceived in the high information condition made participants perceive a higher impact of their donations and higher warm glow. This finding is in line with previous studies associating greater perceived donation impact (Cryder et al., 2008; Cryder and Loewenstein, 2010) and positive feelings associated with a contribution to the cause (Cryder et al., 2008; Cryder and Loewenstein, 2010) with greater tangibility derived from detailed textual information. Furthermore, even though we found no significant direct effect of the condition on the amount donated (ATE), our results showed that higher tangibility increased participants’ motivation to donate and consequently the amount they would hypothetically donate, through the mediating effect of higher perceived donation impact and warm glow. These findings are in line with previous studies showing that perceived donation impact (Cryder et al., 2013; Erlandsson et al., 2014, 2015) and higher positive feelings (Andreoni, 1990; Dickert et al., 2011) mediate the motivation to donate.

As suggested by the two-stage model (Dickert et al., 2011), our results showed that the warm glow had a direct effect on the motivation to donate but not on the amount they were willing to donate. According to this model, the donation process is organized in two stages: Stage 1, i.e., the initial motivation and decision to donate, and Stage 2, i.e., the choice of how much to donate. Each stage is driven by different mechanisms: while the first is driven by emotions directed to the self (e.g., warm glow), the second is driven by emotions directed to others (e.g., empathy). In the present study, however, the perceived impact was found to affect only Stage 1 as warm glow did since no direct effect was detected on the amount donated. Considering that the perceived donation impact is the result of a trade-off between the expected benefits for the recipients and the costs for the donors (Caserotti et al., 2019) and that the latter is weighted more (Rubaltelli and Agnoli, 2012; De Bruyn and Prokopec, 2013; Sussman et al., 2015; Rubaltelli et al., 2020). We can speculate that perceived impact affected only the first stage of the model since it entails more self-oriented emotions similarly to warm glow. Indeed, in line with this speculation, our results showed that participants who perceived higher impact showed also a higher warm glow.

Our findings showed that the indirect effect, and not the direct effect, of the background information of charity appeal can have an effect on charitable donations. Therefore, taking into consideration the pivotal role of tangibility and the precursors of donation, our results can also have potential practical implications. For instance, including high background details in the pictures used for online or printed appeals could be a low-cost expedient that charities can use to boost the effectiveness of their fundraising campaigns. Background information could represent an ethical alternative to the debatable exposure and exploitation of inappropriate and shocking personal images of the victims’ emotional, facial, and physical characteristics to increase appeal’s pervasiveness. Unlike regular businesses, non-profit organizations are generally held to higher ethical standards (Lawry, 1995) and should consider avoiding using the victim’s sorrow in a demeaning way. However, the fact that people are more likely to donate to a hospital that looks already adequately prepared is somewhat disheartening, especially considering the conditions of many realities around the World. Nevertheless, the suggested applications should be taken with caution since further studies (e.g., within-subjects design or field studies) are required to corroborate our results.

Further, the data of the present study were collected in the emergency context of the COVID-19 pandemic. We thus also controlled for the role of COVID-19 risk perception in shaping perceived tangibility and donation behaviors. Participants with a high perception of risk associated with COVID-19 perceived the hospital as more adequately prepared to deal with the medical emergency, their donation as more impactful, and felt a higher warm glow. Consequently, people with higher COVID-19 risk perception showed higher motivation to donate to a COVID-19 relief found and higher stated donation amounts. This result is in line with previous literature suggesting that perceiving COVID-19 as highly risky increases donation for causes related to the ongoing pandemic (Abel et al., 2021; Adena and Harke, 2021). Nevertheless, considering the peculiarity of the COVID-19 emergency (e.g., highly dreadful, very close, and world-spread), it is possible that other elements related to the pandemic (e.g., personal knowledge about the situation of the hospitals, familiarity with the disease) might have affected our results. Thus, future studies should try to replicate and generalize the effect of visual background information also with different types of scenarios and in non-emergency contexts. It is indeed possible that the role of visual background information might be particularly important in increasing tangibility, and in turn generosity, for more distant, both strictly and figuratively, causes (e.g., a medical emergency, unknown in the Western World, in a far country on the other side of the globe). Further, it was recently shown that although COVID-19-related risk perception correlates positively with pandemic-related donations, when people can choose among multiple causes to support, and thus other aspects take over in the assessment, COVID-19 risk perception is no longer significant (Blanco et al., 2021). Future studies should therefore investigate the role of visual background information when different causes are compared jointly.

Moreover, in the present study, we used a picture of a man with his back turned. This choice was made to avoid confounding effects of personal characteristics and facial expressions. Nevertheless, it could be interesting to investigate how detailed information related to the victim and those related to the context might interact and which of the two is effectively more powerful. Furthermore, we could not control how much attention participants actually paid to the visual details manipulated in the pictures’ background. Future studies should thus consider implementing process measures, e.g., eye-tracking tools, that can track attention allocation in specific areas of interest to better understand the effect of similar manipulations.

In addition, we investigated hypothetical rather than actual donation decisions. Although this choice might limit our findings’ generalizability, extant literature showed similarities in the psychological mechanisms behind hypothetical and real contributions (Kogut and Ritov, 2005; Dickert et al., 2016). Nevertheless, future studies should test our model with actual donations to increase its ecological validity. Besides, although donations from a single individual may not be repeated over time, the effect multiplied by the number of people who may be exposed to the charity advertisement makes the result relevant for policy aiming (Funder and Ozer, 2019). However, since this is the first study on this topic, we encourage future studies with bigger samples to corroborate our results.

In conclusion, the present study shows that high background information in charity appeal’s pictures can increase people’s stated generosity through perceived tangibility and the precursors of donation (e.g., perceived impact and warm glow). We think that these first results hold potentially interesting insights from both a theoretical and practical perspective that is worth investigating further.

## Data Availability Statement

The datasets presented in this study can be found in online repositories. The names of the repository/repositories and accession number(s) can be found below: OSF https://osf.io/yw3uz/?view_only=015933180278497998bb1289551a7e12.

## Ethics Statement

Ethical review and approval were not required for the study on human participants in accordance with the local legislation and institutional requirements. The patients/participants provided their written informed consent to participate in this study.

## Author Contributions

MC and MV: conceptualization, formal analysis, visualization, writing—original draft, and writing—review and editing. MM: conceptualization, and writing—review and editing. GP: conceptualization, visualization, writing—original draft, and writing—review and editing. All authors contributed to the article and approved the submitted version.

## Conflict of Interest

The authors declare that the research was conducted in the absence of any commercial or financial relationships that could be construed as a potential conflict of interest.

## Publisher’s Note

All claims expressed in this article are solely those of the authors and do not necessarily represent those of their affiliated organizations, or those of the publisher, the editors and the reviewers. Any product that may be evaluated in this article, or claim that may be made by its manufacturer, is not guaranteed or endorsed by the publisher.
