# Supplementary material for: Look Behind Me! Highly Informative Picture Backgrounds Increase Stated Generosity Through Perceived Tangibility, Impact, and Warm Glow
Source: Front Psychol. 2022 Apr 6;13:800199. doi: 10.3389/fpsyg.2022.800199 (PMC9020366; doi:10.3389/fpsyg.2022.800199)
Supplement: Supplementary file 1 [file Data_Sheet_1.pdf]

frontiers

## *Supplementary Material*

**Supplementary Method 1. Introductory scenario and manipulations in the three experimental conditions.**

**All participants were presented with the following textual scenario followed by one of the three pictures described below:**

*COVID-19 Relief Fund for your local hospital*

*"We need your help! Donate today to the COVID-19 Relief Fund to help meet the emergency needs of your local hospital. Your support will allow our care team to best respond to COVID-19 locally and to ensure the hospital has the funds to quickly adapt to any situation that comes up as the pandemic evolves."*

1) **High information condition**

A caucasian, middle-aged man with brown hair was depicted in a hospital chamber, sitting on a hospital bed facing a large window. The man was wearing a light-green surgical vest and was attached to an I.V. The man was depicted with his back turned. On the left corner of the chamber, a bedside table was depicted with a piece of medical machinery on it. A monitor was also present. On the right corner of the chamber, several types of medical machinery were shown including two monitors, a defibrillator, and a respiratory machine**.**

2) **Low information condition**

A caucasian, middle-aged man with brown hair was depicted in a hospital chamber, sitting on a hospital bed facing a large window. The man was wearing a light-green surgical vest and was attached to an I.V. The man was depicted with his back turned. On the left corner of the chamber, an empty bedside table was depicted. The right corner of the chamber was empty. Except for the I.V, no other medical equipment or machinery was shown in this condition.

3) **No information condition**

A caucasian, middle-aged man with brown hair was depicted sitting on a hospital bed with white sheets. The man was wearing a light-green surgical vest and was depicted with his back turned. The bed was presented without context on a white background, without any kind of medical machinery of any sort.

Supplementary Material

**Supplementary Table 1. Main characteristics of the sample by conditions.**

Descriptive analyses are summarized in frequency tables (frequency or categorical variables and median and InterQuartile Range for continuous variables). Non-parametric test (Kruskal-Wallis test) was computed to compare the distribution across strata given the predominant non-normal distribution of the continuous variable. Categorical variables were compared using chi-squared. Statistical significance was assumed at the 5% level.

| Variable | No information (N = 161) | Low information (N = 156) | High information (N = 157) | *p* |
|---|---|---|---|---|
| **Gender**, n (%) | | | | 0.52 |
| F | 68 (42.2) | 73 (47.1) | 75 (48.1) | |
| M | 93 (57.8) | 82 (52.9) | 81 (51.9) | |
| **Age**, Median (IQR) | 36.0 (16.3) | 35.0 (14.0) | 35.0 (18.0) | 0.71 |
| **Education**, n (%) | | | | 0.23 |
| Low | 15 (9.3) | 25 (16.0) | 28 (17.8) | |
| Medium | 103 (64.0) | 95 (60.9) | 95 (60.5) | |
| High | 43 (26.7) | 36 (23.1) | 34 (21.7) | |
| **Political orientation**, Median (IQR) | 3.0 (8.0) | 2.0 (8.50) | 3.0 (7.0) | 0.58 |
| **Income**, n (%) | | | | 0.71 |
| <20k | 13 (8.2) | 11 (7.1) | 13 (8.3) | |
| 20k-40k | 29 (18.4) | 33 (21.3) | 25 (16.0) | |
| 40k-60k | 57 (36.1) | 45 (29.0) | 51 (32.7) | |
| 60k-80k | 28 (17.7) | 30 (19.4) | 27 (17.3) | |
| 80k-100k | 16 (10.1) | 22 (14.2) | 19 (12.2) | |
| >100k | 15 (9.5) | 14 (9.0) | 21 (13.5) | |
| Prefer not to say | 3 (1.9) | 1 (0.6) | 1 (0.6) | |
| **Insurance**, n (%) | | | | 0.77 |
| No insurance | 17 (10.6) | 18 (11.6) | 21 (13.5) | |
| Private insurance | 52 (32.3) | 48 (30.3) | 39 (25.0) | |
| Public insurance | 66 (41.0) | 69 (44.5) | 74 (46.8) | |
| Both insurance | 26 (16.1) | 21 (13.5) | 23 (14.7) | |

**Note.** Education (Low: school education up to age 18; Medium: bachelor degree; High: Master or other postgraduate qualifications), Political orientations (-10 = "Left wing (liberal)", +10 ="Right wing (conservative)").

**Supplementary Table 2. ANOVA to test the effect of Condition on the Decision to donate.**

We ran a one-way ANOVA to test the effect of Condition (No information = 1, Low information = 2, High information = 3) on the Decision whether to donate or not. Results show that the Decision did not vary across conditions ($F(2, 474) = .15$, $p = .863$). Further, a Tukey multiple comparison test showed no differences between the three conditions ($p = .86$ or higher). We reported the frequencies of choice for the Decision.

| Variable | No information (N = 161) | Low information (N = 156) | High information (N = 157) | *p* |
|---|---|---|---|---|
| **Donation**, n (%) | | | | 0.86 |
| Yes | 128 (79.5) | 123 (78.8) | 121 (77.1) | |
| No | 33 (20.5) | 33 (21.1) | 36 (22.9) | |

**Supplementary Method 2. One-way ANOVA to test the effect of Condition on tangibility.**

We ran a one-way ANOVA to test the effect of Condition (No information = 1, Low information = 2, High information = 3) on our tangibility variables (i.e., Adequacy and Severity of the patient). Results show that Severity of the patient did not vary across conditions, while Adequacy did (see Supplementary Table 2 and Supplementary Table 3). Specifically, post hoc analyses revealed that participants perceived a higher adequacy of the hospital when the background presented high information rather than when it presented low information. In particular, going from a low- to high information increase perceived adequacy with 12%. Nevertheless, the difference in perceived Adequacy between High or Low Information conditions and the No Information condition was not significant (see Supplementary Table 4).

**Supplementary Table 3. ANOVA testing the effect of Condition on the tangibility variables**

| | Sum of Squares | df | Mean Square | F | *p* | $\eta^2$ | Observed power |
|---|---|---|---|---|---|---|---|
| Adequacy | 167.68 | 2 | 83.84 | 3.48 | .032 | .015 | .649 |
| Severity of the patient | 26.55 | 2 | 13.28 | .43 | .650 | .002 | .120 |

**Supplementary Table 4. Descriptive analysis of the tangibility variables per conditions**

| | | N | Mean | SD |
|---|---|---|---|---|
| Adequacy | No information | 161 | 2.24 | 4.91 |
| | Low information | 156 | 1.98 | 5.20 |
| | High information | 157 | 3.36 | 4.60 |
| Severity of the patient | No information | 161 | 2.16 | 5.53 |
| | Low information | 156 | 1.63 | 5.48 |
| | High information | 157 | 1.69 | 5.63 |

**Supplementary Table 5. Post-hoc analysis of the effect Condition on the tangibility variables**

| | | | Mean difference | SE | *p* | 95% CI | |
|---|---|---|---|---|---|---|---|
| | | | | | | LL | UL |
| Adequacy | No information | Vs. Low | .26 | .55 | .884 | -1.04 | 1.56 |
| | | Vs. High | -1.11 | .55 | .108 | -2.41 | .18 |
| | Low information | Vs. No | -.26 | .55 | .884 | -1.56 | 1.04 |
| | | Vs. High | -1.38* | .56 | .036 | -2.68 | -.07 |
| | High information | Vs. No | 1.11 | .55 | .108 | -.18 | 2.41 |
| | | Vs. Low | 1.38* | .56 | .036 | .07 | 2.68 |
| Severity of the patient | No information | Vs. Low | .53 | .62 | .675 | -.94 | 1.99 |
| | | Vs. High | .47 | .62 | .733 | -.99 | 1.93 |
| | Low information | Vs. No | -.53 | .62 | .675 | -1.99 | .94 |
| | | Vs. High | -.06 | .63 | .995 | -1.53 | 1.41 |
| | High information | Vs. No | -.47 | .62 | .733 | -1.93 | .99 |
| | | Vs. Low | .06 | .63 | .995 | -1.41 | 1.53 |
